# The human lower leg muscle pump functions as a flow diverter pump, maintaining low ambulatory venous pressures during locomotion

**DOI:** 10.1016/j.jvsv.2024.101996

**Published:** 2024-10-22

**Authors:** Roman A. Tauraginskii, Fedor Lurie, Sergei Simakov, Rishal Agalarov, Pavel Khramtsov, Maxim Babushkin, Tatiana Gurina, Denis Borsuk

**Affiliations:** a Research Laboratory of Venous Hemodynamics, Phlebocenter LLC, Kaliningrad, Russia; b Jobst Vascular Institute, Toledo, OH; c Division of Vascular Surgery, University of Michigan, Ann Arbor, MI; d Department of Computational Physics, Moscow Institute of Physics and Technology, Moscow, Russia; e Sechenov University, Moscow, Russia; f Clinic of Phlebology "VenoClinica", Yekaterinburg, Russia

**Keywords:** Ambulation, Ambulatory venous pressure, Chronic venous insufficiency, Human locomotion, Muscle pump, Muscle pump failure

## Abstract

**Objective:**

Ambulatory venous pressure (AVP) is the drop of pressure observed in the superficial veins of the lower leg during movement. This phenomenon has been linked to the function of the calf muscle pump (CMP) and the competence of venous valves. Nevertheless, the concept of the CMP function remains controversial. This study aimed to elucidate the association between lower leg muscles activity, changes in pressure in distinct venous segments, and lower extremity arterial blood supply in healthy subjects during various types and intensities of exercise.

**Methods:**

Twelve legs of nine healthy volunteers were enrolled in the study. Continuous pressure (intramuscular vein [IV] and three great saphenous vein [GSV] points) and surface electromyography data (gastrocnemius and anterior tibial [ATM] muscles) were recorded during treadmill walking, running, and plantar flexion exercises. The pressure gradient (ΔP, mmHg) between adjacent points of measurement was calculated. Minute unit power of muscle pump ejection and suction (*N*_*E*_, and *N*_*S*_, MPa/min) were calculated and compared with the arterial blood supply of the lower extremity (LBF, L/min).

**Results:**

ΔP demonstrated a consistent pattern of changes during walking and running. In GSV, the ΔP was observed to be directed from the thigh to the mid-calf (retrogradely) and from the ankle to the mid-calf (anterogradely) throughout the entire stride cycle. However, its value decreased with increasing stride cycle frequency. The dynamics of ΔP between the IV and GSV were as follows: It was directed from the IV to GSV during gastrocnemius contraction and was reversed during anterior tibial muscle contraction and gastrocnemius relaxation (swing phase). LBF, *N*_*E*_, and *N*_*S*_ demonstrated similar exponential growth with increasing stride frequency during walking and running.

**Conclusions:**

During natural locomotion, the muscle pump acts as a flow diverter pump, redirecting the flow of blood from the superficial veins to the intramuscular veins via the perforating veins. During ambulation, the pressure in the superficial venous network depends upon the capacity of the muscle pump to provide output that matches the changes in arterial blood flow.

**Article Highlights**

• **Type of Research:** Prospective experimental study
• **Key Findings:** An absence of pressure gradient directed from the calf to the thigh in the great saphenous vein was observed. Instead, pressure gradient directed from the great saphenous vein to intramuscular veins was verified during anterior tibial muscle contraction and gastrocnemius relaxation.
• **Take Home Message:** The calf muscle pump is a two-component mechanism that involves the synergistic work of antagonist calf muscle groups, namely the anterior and posterior muscles, rather than relying solely on the posterior muscle group. Blood from the superficial veins of the lower leg primarily drains towards the intramuscular veins during exercise.

Ambulatory venous pressure (AVP) is regarded as the “gold standard” functional test estimating both hemodynamic abnormalities and the severity of chronic venous disease (CVD).^1^ In a standing position, venous pressure is determined mainly by hydrostatic pressure, and at the lower leg and foot levels, it ranges on average from 60 to 90 mmHg, depending on the distance from the measurement point to the right atrium.^2^, ^3^, ^4^, ^5^ In healthy subjects, during an exercise such as walking, tiptoe movements, knee bending, etc, venous pressure in the superficial venous network drops significantly by more than 60% relative to the standing still value, with a mean minimal value of 30 to 40 mmHg.^6^, ^7^, ^8^, ^9^, ^10^, ^11^, ^12^, ^13^, ^14^, ^15^ This phenomenon, designated as AVP, has been attributed to the pumping action of the calf muscles (CMP) and the competency of the venous valves in preventing retrograde of blood flow during the period of muscular relaxation.^2^^,^^3^^,^^7^^,^^8^^,^^16^ However, the aforementioned concept does not elucidate the observed variation in AVP values between diverse movement types (plantar flexions, walking in a spot, walking or running on a treadmill, etc.) done by the same healthy subject,^3^^,^^17^, ^18^, ^19^, ^20^, ^21^ nor does it account for the variation in AVP between the same movement type performed by the same subject in different environmental temperatures (cold vs warm).^17^^,^^22^

We hypothesized that in healthy subjects, the drop and maintenance of venous pressure in the superficial veins during exercise, at relatively low values (AVP ≤30 mmHg), is a consequence of the power generated by CMP having to be closely matched with the changes in arterial blood supply correlated to exercise intensity. During natural locomotion, the muscle pump is responsible for redirecting blood flow from the superficial veins to the intramuscular veins (IVs), acting as a flow diverter pump. This is achieved through the coordinated activity of the anterior and posterior calf muscle groups, as well as the ankle joint.

This study aimed to investigate the real-time relationship between lower extremity arterial blood supply, gastrocnemius and anterior tibial muscle activity, and pressure changes in different venous segments in healthy subjects during exercises with specific types of biomechanics and intensity.

## Methods

The methods employed were outlined extensively in the preceding study.^23^ The study conformed to the Declaration of Helsinki. The local ethics committee of Surgut State University approved the study (protocol #22 of 02.17.2021). The participants were fully informed of the study protocol and risk of potential complications (thrombosis, bleeding, infections etc). The volunteers provided written consent to engage in the study. There were not any complications in the study. The same sample (12 lower extremities) of nine healthy volunteers (seven men and two women) was enrolled in the study.^23^ Inclusion criterion was a suitable lower leg venous anatomy allowing catheterization of large intramuscular vein of the gastrocnemius muscle without muscle damage. Exclusion criteria included clinical signs (C_1-6_) according to the clinical, etiologic, anatomic, and pathological (CEAP) classification^24^ of CVD; ultrasound-detectable reflux or obstruction of superficial, perforating, and/or deep veins; previous venous surgery; sclerotherapy; impaired ankle, knee, or hip joint mobility; and ankle-brachial index <0.9. The subjects’ anthropometrics (mean ± standard deviation [SD]) were: age, 32.8 ± 5 years; height, 177 ± 5 cm; weight, 79.7 ± 16 kg; body mass index, 25 ± 4 kg/m^2^; and calf circumference, 38.2 ± 3.3 cm.

### Experiment preparation

**Fig 1:**
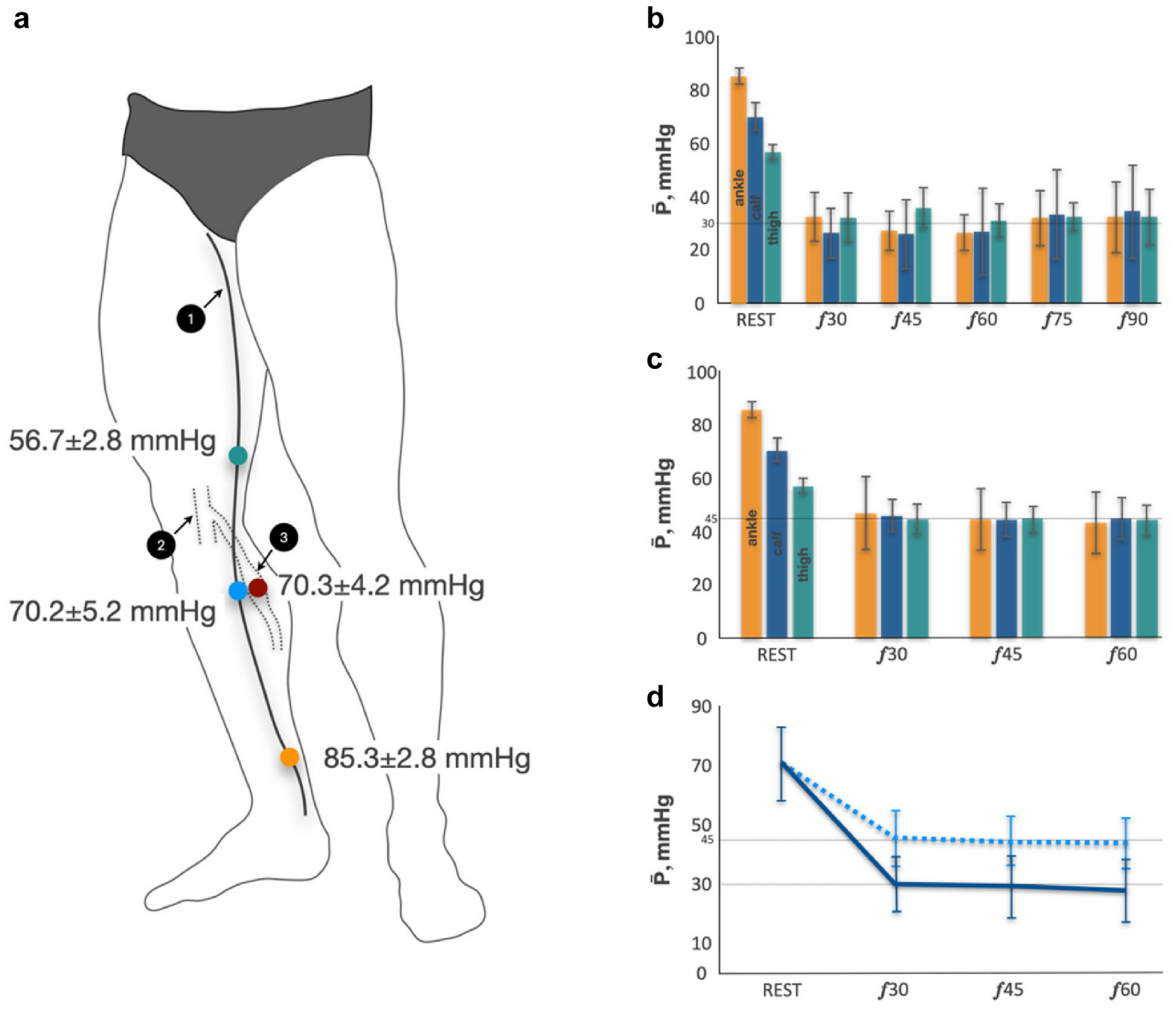
**A,** Pressure data at a standing still position (mean and standard deviation [SD]). 1, great saphenous vein (GSV); 2, popliteal vein; 3, intramuscular vein (IV) of gastrocnemius. *Orange point*, GSV at ankle level; *blue point*, GSV at proximal calf level; *green point*, GSV at mid-distal thigh level; *red point*, IV at proximal calf level. **B**, Cycle time-average pressure during walking and running (mean and SD). *Orange column*, cycle time-average pressure at GSV ankle level; *blue column*, cycle time-average pressure at GSV proximal calf level; *green column*, cycle time-average pressure at GSV mid-distal thigh level. *Rest*, standing still position; *F*, stride frequency per minute. **C,** Cycle time-average pressure during plantar flexion (mean and SD). *Orange column*, cycle time-average pressure at GSV ankle level; *blue column*, cycle time-average pressure at GSV proximal calf level; *green column*, cycle time-average pressure at GSV mid-distal thigh level. *Rest*, standing still position; *F*, flexions frequency per minute. **D,** Cycle time-average pressure averaged by three GSV points (mean and SD). *Blue dotted line*, cycle time-average pressure in GSV during plantar flexion; *blue solid line*, cycle time-average pressure in GSV during walking. *Rest*, standing still position; *F*, movement frequency per minute.

Subjects came to the laboratory at 10:00 AM and were instructed not to eat or drink coffee or tea and to avoid physical exercise for 2 hours before the experiment. Then, volunteers rested in a lying position for around 30 to 40 minutes while catheterization of the target veins was done under local anesthesia using ultrasound guidance. Three intravenous 18-gauge catheters filled with heparinized saline were placed in the great saphenous vein (GSV) at the ankle [GSV_ANKLE_], upper third of the calf [GSV_CALF_], and mid-distal thigh [GSV_THIGH_] levels. A 20-cm-long 5 French catheter (Balton Sp.z.o.o.) filled with heparinized saline was placed in the large IV of the gastrocnemius medial aspect (IV_CALF_). The IV was deemed suitable for catheterization if it had a connection with the superficial venous system via a perforating vein of an appropriate size and anatomy, allowing for catheterization without muscle damage.^23^ The intramuscular catheter was adjusted to the level of the GSV_CALF_ catheter. All catheterization points are presented in Fig 1, *A*. The catheters were secured by cannula retention dressing. Pressure transducers (TruWave 3 cc/60 PX260, Edwards Lifescience Services GmbH) connected to the catheters were secured on the lateral aspect of the calf and thigh at the same level as the catheters. A multi-channel blood pressure analysis system, “Angioton” (BIOSOFT-M Ltd), was used for continuous pressure monitoring. A surface electromyography (sEMG) was used to measure an activation of gastrocnemius medial aspect (GCM) and anterior tibial muscle (ATM) during each exercise bout (Callibri Group Ltd). The sEMG probes were connected to the system software installed on a personal computer by Bluetooth. The software displayed sEMG data as real-time graphs.

### Experiment protocol

The volunteers consistently completed the following exercise tests. First, treadmill (Physioline TNX TOUCH) walking with a frequency of 30, 45, and 60 stride cycles/min. The stride cycle is the time from the heel strike of one foot to the next heel strike by the same foot; during locomotion, the number of stride cycles is one-half of the gait cycles (steps).^25^ Second, treadmill running with 75 and 90 strides/min. Third, plantar flexions in a standing position with 30, 45, and 60 flexions/min. During the plantar flexion test, subjects stood on the treadmill while holding onto its frame. Every exercise bout lasted 1 minute and was separated by 10 minutes of rest. In the pauses between exercise bouts, each catheter was flushed with heparinized normal saline. The laboratory room temperature was adjusted to the thermoneutral zone 21 °С to 23 °С.

The blood volume flow rate in the common femoral artery of the studied leg (LBF) was calculated as the product of the cross-sectional area (CSA) received from the ultrasound B-mode image, the time-average velocity (TAMEAN), and the heart rate (HR), both measured in a longitudinal plane and averaged over a 12-sec Doppler scan (LBF = CSA × TAMEAN × HR, liter/min). The ultrasound measurements were taken in a standing still position once before the exercise session and as soon as the subject stops after each exercise bout. During exercise, the pressure and sEMG data were recorded continuously. Each exercise set was recorded by a video camera embedded in a smartphone (iPhone 11 Pro, Apple Inc) using 1080 pixels in a 60-frames/sec format for the ensuing evaluation of the stride and plantar flexion cycles using frame-by-frame analysis of the videos.^26^^,^^27^

### Data processing and analysis

Because walking, running, and plantar flexions are cyclic processes, the time-pressure curves of 10 subsequent strides or flexions of each subject were reinterpolated to a uniform grid with constant time step and then averaged to either single stride or plantar flexion cycle for the ensuing data comparison. To avoid observer bias, the data analysis (the time of stride and plantar flexion cycle events, pressure, and sEMG parameters) was performed using 10 consecutive strides (movements) in the middle of each exercise bout. The cycle time-averaged pressure (mean pressure [P], mmHg) was calculated for each GSV point for each exercise set. The pressure gradient (ΔP, mmHg) between adjacent points of measurement was calculated as $P_{vein1}-P_{vein2}$ for each time moment of either the stride or plantar flexion cycle. Then, Δ*P* values were plotted on a graph for each type of exercise bout. Four ΔP curves were plotted: ΔP _RED_ (IV_CALF_ minus GSV_CALF_), ΔP _WHITE_ (IV_CALF_ minus GSV_ANKLE_), ΔP _ORANGE_ (GSV_CALF_ minus GSV_THIGH_), and ΔP _BLUE_ (GSV_ANKLE_ minus GSV_CALF_). The average minute unit power of muscle pump was calculated as *N*_*E*_ *= m × N*_*E*_ and *N*_*S*_ *= m × N*_*S*_ (Mpa/min) for each type of exercise, where *N*_*E*_ and *N*_*S*_ are cycle-averaged unit power of ejection and suction, respectively, and *m* is the number of either stride or flexion cycles per minute. For more detailed description of the muscle pump unit power calculation, please refer to the Supplementary Appendix (online only) and the Supplementary Fig (online only).

The plantar flexion exercise was included in the study protocol to investigate the importance of a synergistic work of antagonist calf muscles. This refers to the coordinated action of calf muscles, particularly the ATM contraction and gastrocnemius relaxation, which induces rotation of the ankle joint. This is absent during plantar flexion but present during normal ambulation. Another distinction between these types of movements is the centrifugal acceleration of blood during the swing of the leg. This should result in an increase in venous pressure to some extent.

### Statistical analysis

Means and standard deviations were used to describe the continuous variables and their mathematical analysis. Nonparametric analyses were performed for statistical evaluation of the data (*N*_*E*_ and *N*_*S*_ Mpa/cycle, *N*_*E*_ and *N*_*S*_ Mpa/min, LBF L/min, and P mm Hg). The Friedman test and post hoc paired Mann-Whitney-Wilcoxon test with the Benjamini-Yekutieli correction were used to compare the parameters between different exercise frequencies. The Mann-Whitney-Wilcoxon test was used to compare the parameters between different types of exercise at the corresponding frequency. For multiple comparisons, the Benjamini-Yekutieli correction was used. The correlation analysis used the Spearman rank correlation coefficient. Statistical significance was defined as *P* < .05. The analysis was performed with Statistica 13 (Statsoft Inc), R Language 4.3 (R Foundation), and Rstudio 2023.03 (Posit Software, PBC).

## Results

### Ambulatory venous pressure

Venous pressure in all GSV points of measurement was different in the standing still position, but it decreased to approximately the same value during exercise (Fig 1). There was no significant difference in cycle time-averaged pressure (P, mmHg) between three GSV points at any frequency used during walking, running, or plantar flexion (Fig 1, *B* and *C*). In addition, there was no difference in *P* values at all GSV points between the used exercise frequencies. However, the P at each GSV point was statistically significantly higher during the plantar flexion exercise compared with the corresponding frequency of the walking exercise. Fig 1, *D* shows that P averaged over three GSV points was 50% higher during plantar flexion than during walking at any of exercise frequency.

### Pressure changes across stride and plantar flexion cycles

**Fig 2:**
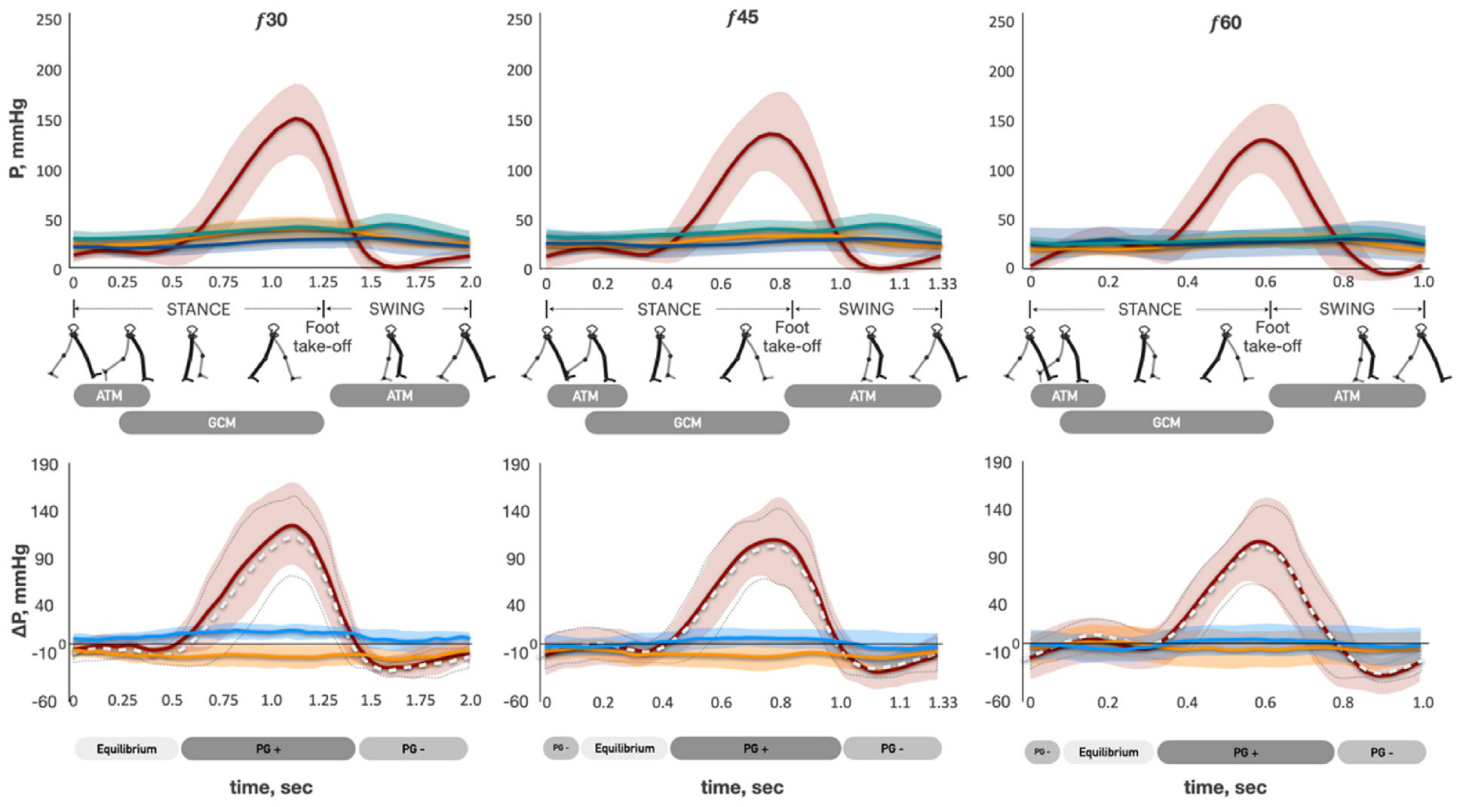
Pressure and pressure gradient changes across the stride cycle of ambulation. The top row is pressure curves at different stride cycle frequencies. *P*, pressure; *solid red curve*, mean pressure of the intramuscular vein of gastrocnemius (IV_CALF_); *light red area*, standard deviation of mean IV_CALF_ pressure; *solid green curve*, mean pressure of great saphenous vein (GSV) at mid-distal thigh level (GSV_THIGH_); *light green area*, standard deviation (SD) of mean GSV_THIGH_ pressure; *solid blue curve*, mean pressure of GSV at proximal calf level (GSV_CALF_); *light blue area*, SD of mean GSV_CALF_ pressure; *solid orange curve*, mean pressure of GSV at ankle level (GSV_ANKLE_); *light orange area*, SD of mean GSV_ANKLE_ pressure. *f*, stride frequency per minute; *ATM*, anterior tibial muscle; *GCM*, gastrocnemius muscle. *Gray boxes*, timeline of ATM and GCM activation (surface electromyography [sEMG] data). The bottom row is pressure gradient curves at different stride cycle frequencies. *ΔP*, pressure gradient; *Solid red curve*, mean of ΔP between IV_CALF_ and GSV_CALF_ (ΔP_RED_ = P IV_CALF_ − P GSV_CALF_); *light red area*, SD of ΔP_RED_; *dotted white curve*, mean of ΔP between IV_CALF_ and GSV_ANKLE_ (ΔP_WHITE_ = P IV_CALF_ − P GSV_ANKLE_); *area bounded by dotted gray line*, SD of ΔP_WHITE_; *solid blue curve*, mean pressure of ΔP between GSV_ANKLE_ and GSV_CALF_ (ΔP_BLUE_ = P GSV_ANKLE_ − P GSV_CALF_); *light blue area*, SD of ΔP_BLUE_; *solid orange curve*, mean pressure of ΔP between GSV_CALF_ and GSV_THIGH_ (ΔP_ORANGE_ = P GSV_CALF_ − P GSV_THIGH_); *light orange area*, SD of ΔP_ORANGE_; *light boxes “Equilibrium,”* timeline of negligible ΔP between IV and GSV; *light gray boxes “ΔP−,”* timeline of negative ΔP directed from GSV to IV; *dark gray boxes “ΔP+,”* timeline of positive ΔP directed from IV to GSV.

**Fig 3:**
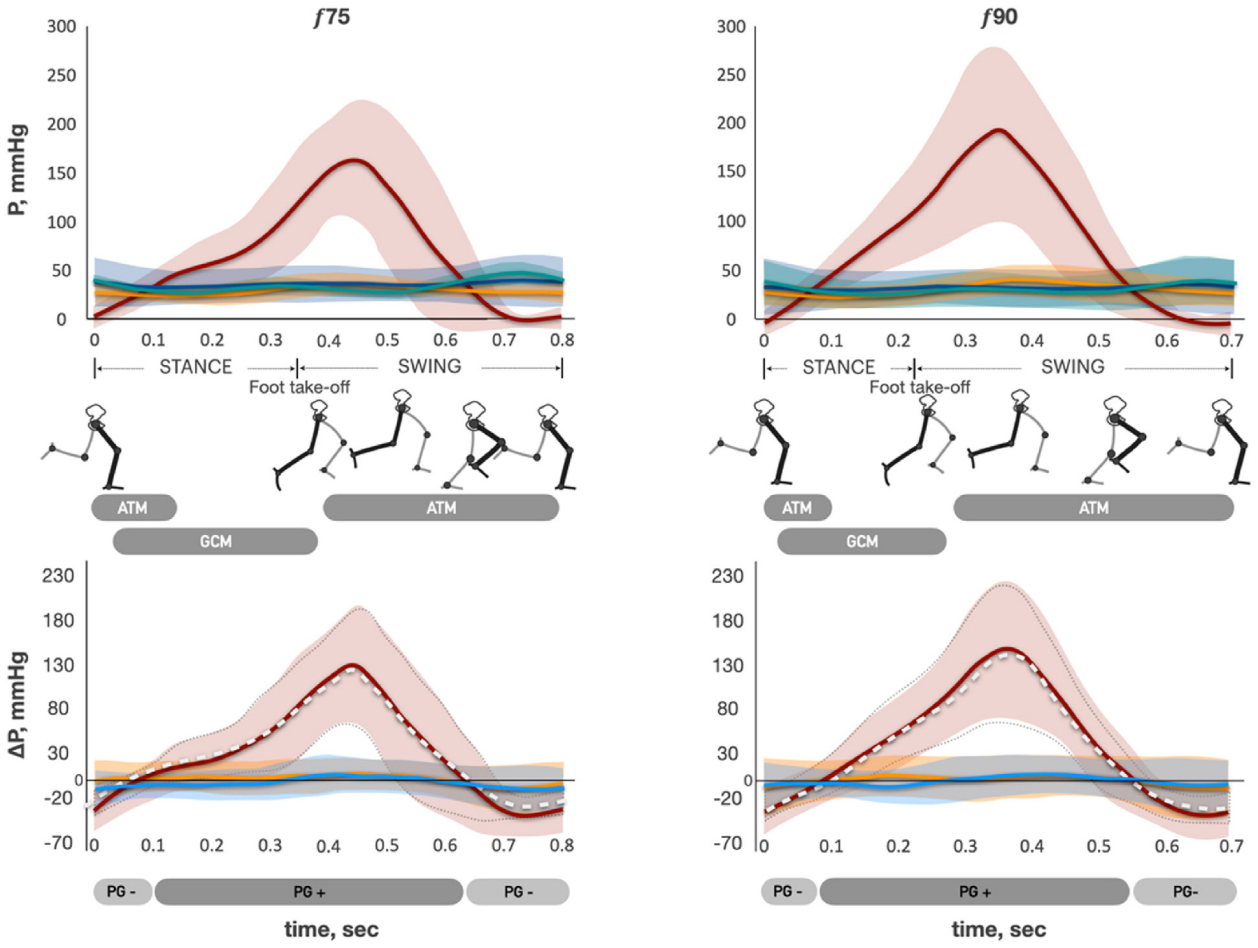
Pressure and pressure gradient changes across the stride cycle of running. The *top row* is pressure curves at different stride cycle frequencies. *P*, pressure; *solid red curve*, mean pressure of the intramuscular vein of gastrocnemius (IV_CALF_); *light red area*, standard deviation (SD) of mean IV_CALF_ pressure; *solid green curve*, mean pressure of GSV at mid-distal thigh level (GSV_THIGH_); *light green area*, SD of mean GSV_THIGH_ pressure; *solid blue curve*, mean pressure of GSV at proximal calf level (GSV_CALF_); *light blue area*, SD of mean GSV_CALF_ pressure; *solid orange curve*, mean pressure of GSV at ankle level (GSV_ANKLE_); *light orange area*, SD of mean GSV_ANKLE_ pressure. *f*, stride frequency per minute; *ATM*, anterior tibial muscle; *GCM*, gastrocnemius muscle. *Gray boxes*, timeline of ATM and GCM activation (surface electromyography [sEMG] data). The bottom row is pressure gradient curves at different stride cycle frequencies. *ΔP*, pressure gradient; *solid red curve*, mean of ΔP between IV_CALF_ and GSV_CALF_ (ΔP_RED_ = P IV_CALF_ − P GSV_CALF_); *light red area*, SD of ΔP_RED_; *dotted white curve*, mean of ΔP between IV_CALF_ and GSV_ANKLE_ (ΔP_WHITE_ = P IV_CALF_ − P GSV_ANKLE_); *area bounded by dotted gray line*, SD of ΔP_WHITE_; *solid blue curve*, mean pressure of ΔP between GSV_ANKLE_ and GSV_CALF_ (ΔP_BLUE_ = P GSV_ANKLE_ − P GSV_CALF_); *light blue area*, SD of ΔP_BLUE_; *solid orange curve*, mean pressure of ΔP between GSV_CALF_ and GSV_THIGH_ (ΔP_ORANGE_ = P GSV_CALF_ − P GSV_THIGH_); *light orange area*, SD of ΔP_ORANGE_; *light gray boxes “ΔP−,”* timeline of negative ΔP directed from GSV to IV; *dark gray boxes “ΔP+,”* timeline of positive ΔP directed from IV to GSV.

**Fig 4:**
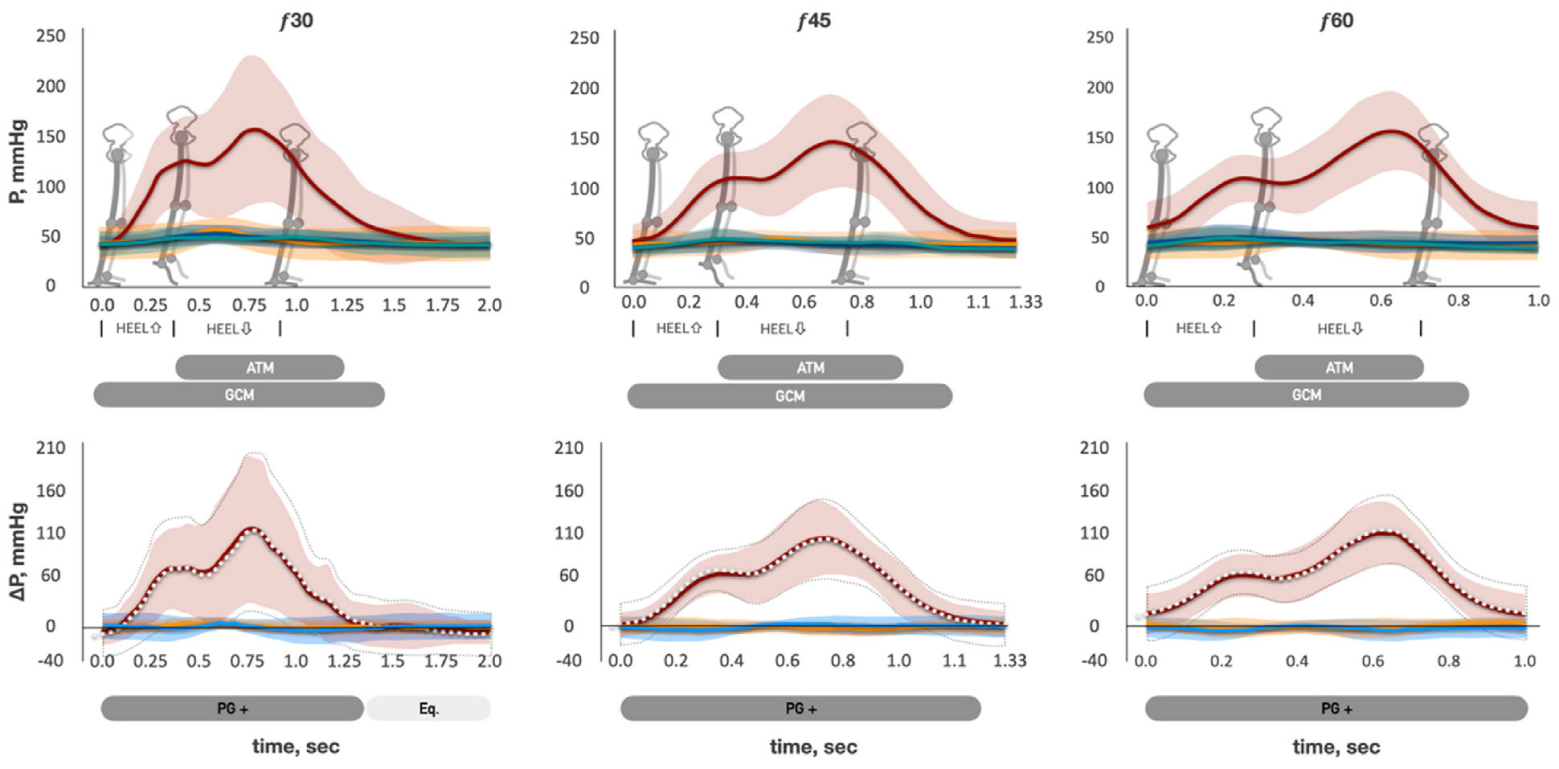
The pressure and pressure gradient changes across the plantar flexion cycle. The top row is pressure curves at different stride cycle frequencies. *P*, pressure; *solid red curve*, mean pressure of the intramuscular vein of gastrocnemius (IV_CALF_); *light red area*, standard deviation (SD) of mean IV_CALF_ pressure; *solid green curve*, mean pressure of GSV at mid-distal thigh level (GSV_THIGH_); *light green area*, SD of mean GSV_THIGH_ pressure; *solid blue curve*, mean pressure of GSV at proximal calf level (GSV_CALF_); *light blue area*, SD of mean GSV_CALF_ pressure; *solid orange curve*, mean pressure of GSV at ankle level (GSV_ANKLE_); *light orange area*, SD of mean GSV_ANKLE_ pressure. *f*, stride frequency per minute; *ATM*, anterior tibial muscle; *GCM*, gastrocnemius muscle. *Gray boxes*, timeline of ATM and GCM activation (surface electromyography [sEMG] data). The *bottom row* is pressure gradient curves at different stride cycle frequencies. *ΔP*, pressure gradient; *solid red curve*, mean of ΔP between IV_CALF_ and GSV_CALF_ (ΔP_RED_ = P IV_CALF_ − P GSV_CALF_); *light red area*, SD of ΔP_RED_; *dotted white curve*, mean of ΔP between IV_CALF_ and GSV_ANKLE_ (ΔP_WHITE_ = P IV_CALF_ − P GSV_ANKLE_); *area bounded by dotted gray line*, SD of ΔP_WHITE_; *solid blue curve*, mean pressure of ΔP between GSV_ANKLE_ and GSV_CALF_ (ΔP_BLUE_ = P GSV_ANKLE_ − P GSV_CALF_); *light blue area*, SD of ΔP_BLUE_; *solid orange curve*, mean pressure of ΔP between GSV_CALF_ and GSV_THIGH_ (ΔP_ORANGE_ = P GSV_CALF_ – P GSV_THIGH_); *light orange area*, SD of ΔP_ORANGE_. *Light boxes “Equilibrium,”* timeline of negligible ΔP between IV and GSV; *dark gray boxes “ΔP+,”* timeline of positive ΔP directed from IV to GSV.

The time-dependent pressure curves of stride (walking and running) and plantar flexion cycles are presented in Fig 2, Fig 3, and 4 (*upper graphs*), respectively. The shape of the pressure curve was similar at all points IV_CALF_, GSV_CALF_, GSV_ANKLE_, and GSV_THIGH_ for particular types of exercise independent from the exercise frequency or studied subject. The amplitude of pressure in the IV was substantially greater than the pressure amplitude in all GSV points for all exercises. The pressure amplitude in the GSV gradually decreases with the increase in stride or plantar flexion frequency. Thus, the curves of mean values almost converge at 60, 75, and 90 strides or 45 and 60 flexions/min. The observed shape of IV_CALF_ pressure was significantly different between the three types of exercise. There was no pressure “plateau” at the beginning of the stance phase during running, unlike with walking (Figs 2 and 3). The pressure of the plantar flexion cycle had two substantial consecutive peaks. The first peak corresponded to gastrocnemius concentric contraction (an increase in tension with muscle shortening) when the heel was raised (Fig 4). A second, even higher peak was observed when the gastrocnemius eccentrically contracted (an increase in tension with muscle lengthening) while the heel was going down. When the heel had already been put down, the gastrocnemius continued contracting until the body returned to a posture perpendicular to the ground. The most pronounced change in pressure took place after a heel had already been grounded. The minimum IV_CALF_ pressure (P_min_) during plantar flexion cycles was significantly higher than the P_min_ of the corresponding stride (walking) cycles (f_30_ 42.1 ± 11.6 vs 1.9 ± 3.8 mmHg; *P* < .0001; f_45_ 46.1 ± 16.5 vs 0.3 ± 4.1 mmHg; *P* < .0001; f_60_ 57.2 ± 24.1 vs −4.6 ± 3.5 mmHg; *P* < .0001, respectively). There was no difference in the pattern of pressure changes between right (#4) and left (#8) legs during stride or plantar flexion cycles.

### Pressure gradient changes across stride and plantar flexion cycles

We analyzed the pressure difference (ΔP) between the pairs of adjacent points of measurement throughout the stride and plantar flexion cycles (see the lower graphs in Figs 2, 3, and 4). ΔP _RED_ curve represents the changes in the pressure difference between IV_CALF_ and GSV_CALF_, mmHg. The ΔP _WHITE_ represents the changes in the pressure difference between IV_CALF_ and GSV_ANKLE_, mmHg. ΔP _RED_ and ΔP _WHITE_ have similar shapes during walking, running, and plantar flexion exercise. The difference between ΔP _RED_ and ΔP _WHITE_ decreases with an increase in exercise frequency. Walking at 30 strides/min (Fig 2): During the first one-half of the stance phase (before heel rise), there was no significant ΔP between IV and GSV (curves are close to zero-line, “equilibrium phase”). Then, when the heel starts rising and gastrocnemius concentric contraction begins, ΔP rapidly grows (ΔP is directed from IV to GSV, “ΔP-positive phase”). The highest ΔP was observed at the end of gastrocnemius concentric contraction (end of stance phase). ΔP starts sharply decreasing at the beginning of the swing phase (the moment of foot take-off) when anterior tibial muscle launches the concentric contraction, and the gastrocnemius is already relaxed. It rapidly becomes negative (ΔP is directed from GSV to IV, “ΔP-negative phase”) and reaches its minimum value around the middle of the swing phase. Then, in the remaining swing phase, ΔP slowly comes to the zero-line. Further, an increase in the walking frequency (45 and 60 strides min^−1^) was characterized by a slightly higher ΔP change around the zero-line during the equilibrium phase. Because stride cycle duration decreases (f 30 2.0 ± 0.04 seconds, f 45 1.33 ± 0.02 seconds, f 60 1.0 ± 0.01 seconds, f 75 0.8 ± 0.01 seconds, and f 90 0.66 ± 0.01 seconds) with an increase in its frequency, the absolute time of all three phases decreases as well (Figs 2, 3, and 4). A feature of running is the absence of the equilibrium phase (Fig 3). During the plantar flexion test, there was no ΔP-negative phase; the equilibrium phase was brief at 45 flexions/min and absent at 60 flexions/min (Fig 4).

The ΔP_BLUE_ curve (pressure difference between GSV_ANKLE_ and GSV_CALF_, mmHg) always has positive values during the stride cycle at 30 strides/min. This means that ΔP is directed cranially from the ankle to the calf. The curve approaches the zero-line with the increase in frequency (45, 60, 75, and 90 strides/min). The ΔP_ORANGE_ curve (P GSV_CALF_ minus P GSV_THIGH_, mmHg) always has negative values in the stride cycle at 30 strides/min. This means that ΔP is directed retrogradely from the thigh to the calf. The curve gradually gets closer to zero with the increase in stride frequency (45 and 60 strides/min) and becomes almost zero at 75 and 90 strides/min.

### Unit power of muscle pump

**Fig 5:**
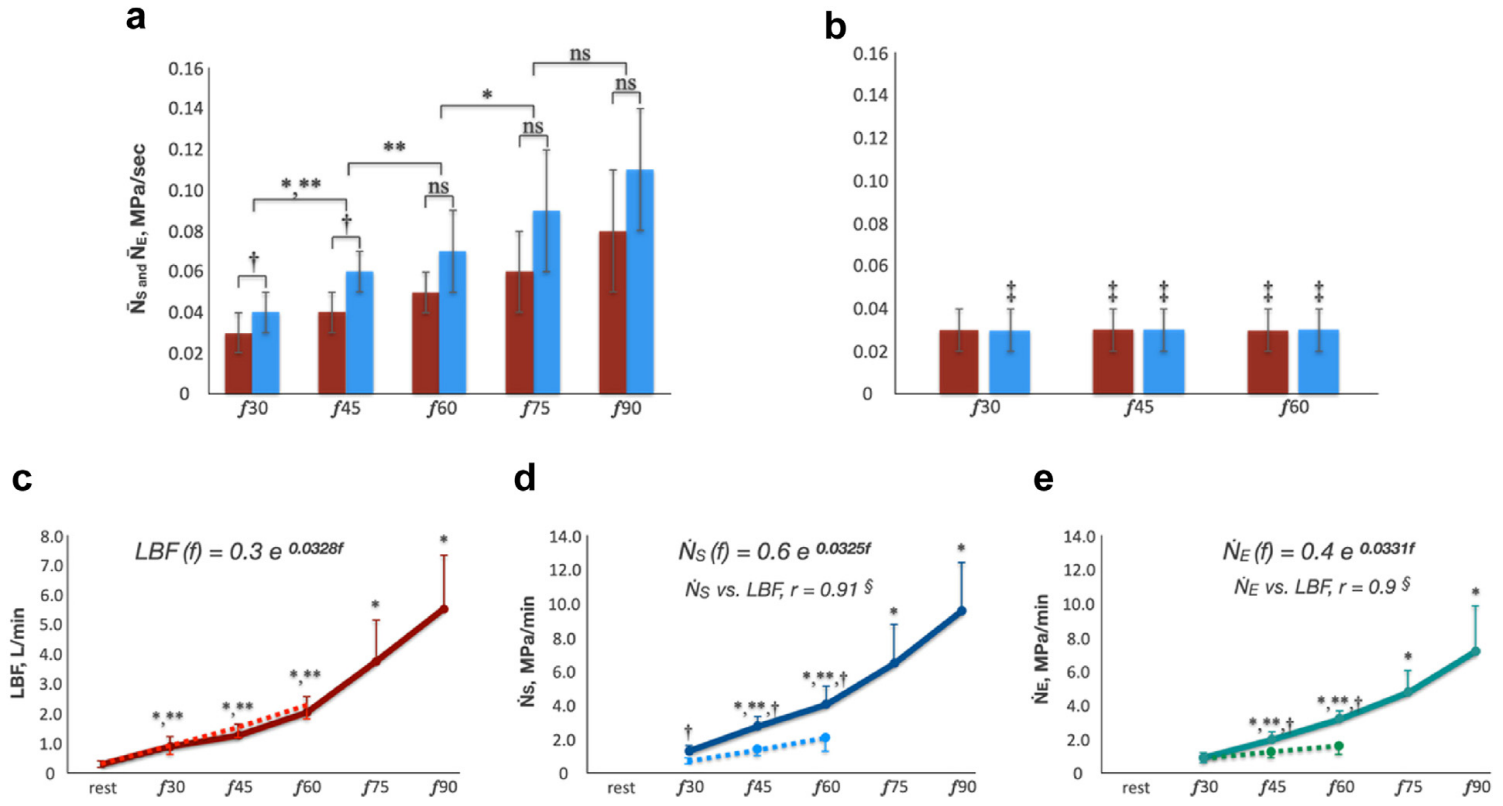
**A,** Average unit power of calf muscle pump (CMP) generated by single stride cycle. **B,** Average unit power of CMP generated by single plantar flexion cycle. *Red column*, cycle-averaged unit power of muscle pump ejection (**N**_**E**_); *blue column*, cycle-averaged unit power of muscle pump suction (**N**_**S**_). *F,* frequency per minute. ∗ Statistically significant difference between N_S_ at different stride frequencies (*P* < .05, Friedman test and post hoc paired Mann-Whitney-Wilcoxon with Benjamini-Yekutieli correction for the multiple comparisons, f45 vs f30, f60 vs f45, f75 vs f60, f90 vs f 75). ∗∗Statistically significant difference between N_E_ at different stride frequencies (*P* < .05, Friedman test and post hoc paired Mann-Whitney-Wilcoxon with Benjamini-Yekutieli correction for the multiple comparisons, f45 vs f30, f60 vs f45, f75 vs f60, f90 vs f 75). ^**†**^Statistically significant difference between N_S_ and N_E_ at corresponding frequencies (*P* < .05, Mann-Whitney-Wilcoxon test with Benjamini-Yekutieli correction for the multiple comparisons). ^‡^Statistically significant difference between the parameters obtained during walking and plantar flexion exercises at corresponding frequencies (*P* < .05, Mann-Whitney-Wilcoxon test with Benjamini-Yekutieli correction for the multiple comparisons, f30 vs f30, f45 vs f45, and f60 vs f60). *Ns*, Not significant (*P* > .05). **C,** Changes in volume flow rate in the common femoral artery (LBF). *Solid red line*, LBF changes during walking and running tests; *dotted red line*, LBF changes during plantar flexion tests. **D,** Changes in minute unit power of calf muscle pump suction (N_S_). *Solid blue line*, *N*_*S*_ changes during walking and running tests; *dotted blue line*, *N*_*S*_ changes during plantar flexion tests. **E,** Changes in minute unit power of CMP ejection (N_E_). *Solid green line*, *N*_*E*_ changes during walking and running tests; *dotted green line*, *N*_*E*_ changes during plantar flexion tests. ∗Statistically significant difference between the parameters (LBF, NS, and NE) at different stride frequencies (*P* < .05, Friedman test and post hoc paired Mann-Whitney-Wilcoxon with Benjamini-Yekutieli correction for multiple comparison, f45 vs f30, f60 vs f45, f75 vs f60, f90 vs f 75). ∗∗Statistically significant difference between the parameters (LBF, N_S_, and N_E_) at different plantar flexion frequencies (*P* < .05, Friedman test and post hoc paired Mann-Whitney-Wilcoxon with Benjamini-Yekutieli correction for multiple comparison, f45 vs f30, f60 vs f45, f75 vs f60, f90 vs f 75). ^**†**^Statistically significant difference between the parameters (LBF, N_S_, and N_E_) obtained during walking and plantar flexion exercises at corresponding frequencies (*P* < .05, Mann-Whitney-Wilcoxon test with Benjamini-Yekutieli correction for the multiple comparisons, f30 vs f30, f45 vs f45, and f60 vs f60.

Fig 5 demonstrates the changes in the average unit power of muscle pump suction (*N*_*S*_) and ejection (*N*_*E*_) generated by a single stride or plantar flexion cycle. *N*_*S*_ and *N*_*E*_ statistically significantly linearly increased with the increase in stride frequency (Fig 5, *A*). Both parameters remained unchanged with the increase in plantar flexion frequency (Fig 5, *B*). The parameters were statistically significantly higher during walking compared with plantar flexion for the corresponding frequencies (Fig 5, *A* and *B*).

Arterial blood flow (LBF, liter/min) and average unit power per minute (*N*_*E*_ and *N*_*S*_, Mpa/min) are presented in Fig 5, *C*, *D*, and *E*. LBF, *N*_*E*_, and *N*_*S*_ all exhibited similar exponential growth with the increase in stride frequency during walking and running. Exponential fitting of the functions LBF(f), $\dot{NS}\left(f\right)$, and $\dot{NE}\left(f\right)$ using the least squares method (LSM) produced the following results:

$${LBF\left(f\right)=0.3e}^{0.0328f}\;{,NS\left(f\right)=0.6e}^{0.0325f}{,NE\left(f\right)=0.4e}^{0.0331f}$$

We observe that the power coefficients are very close in all cases. Thus, we conclude that $\dot{NS}\approx 2LBF$ and $\dot{NE}\approx 1.33LBF$ for all values of frequency f. The LSM and input errors explain the difference between the power coefficients. A strong connection between the parameters was confirmed by correlation analysis (*N*_*S*_ vs LBF r = 0.91, *N*_*E*_ vs LBF r = 0.9; *P* < .0001). The increase in LBF was comparable during plantar flexion and walking exercises. The increase in *N*_*E*_ and *N*_*S*_ was significantly lower.

## Discussion

The main finding of the present study is the establishment of a pattern of pressure correlation between the IV of the gastrocnemius and the GSV, as well as between different levels of the GSV during locomotion and its changes in relation to the biomechanics of movement and the lower extremity arterial blood supply. We describe this pattern by the pressure gradient (mmHg), which changes throughout the stride and plantar flexion cycles. The pattern has the following features. Firstly, in GSV, the pressure gradient was directed from the thigh to the mid-calf (retrogradely) and from the ankle to the mid-calf (anterogradely) throughout the entire stride cycle. However, its value decreased with increasing stride cycle frequency (Figs 2 and 3). Second, a horizontal pressure gradient was observed between the IV and the GSV. Its direction was determined by the coordinated activity of antagonist calf muscles. Its value was considerably higher than the value of pressure gradient between any of the GSV levels (Figs 2 and 3). The cycle time-averaged pressure (P, mmHg) in the GSV did not increase as exercise intensity progressively increased (Fig 1, *B* and *C*). We introduced new parameters to evaluate CMP effectiveness, such as the unit power of muscle pump ejection and suction. These parameters for the single stride cycle (*N*_*E*_ and *N*_*S*_) increased linearly with the increase in stride frequency (Fig 5, *A*). The average minute unit power of the muscle pump (*N*_*E*_ and *N*_*S*_) increased exponentially with the increase in stride frequency and was closely linked to the increased arterial blood supply of the lower extremity for walking and running (Fig 5, *C-E*).

Arterial flow to the working muscles has been demonstrated to occur during their relaxation phase.^28^^,^^29^ Additionally, the intramuscular venous network should be filled with blood from the superficial venous network during muscle relaxation corresponding to the swing phase of the stride cycle. Almen and Nylander^12^ and Ludbrook^13^ have conclusively demonstrated this in the case of plantar flexion with a 2- to 6-second pause between contractions. We observed the pressure gradient directed from either of the GSV levels to IV (ΔP-negative phase) from the middle swing phase until the loading response phase of the next stride cycle. Thus, we confirm the same hypothesis for actual ambulation as well.

**Fig 6:**
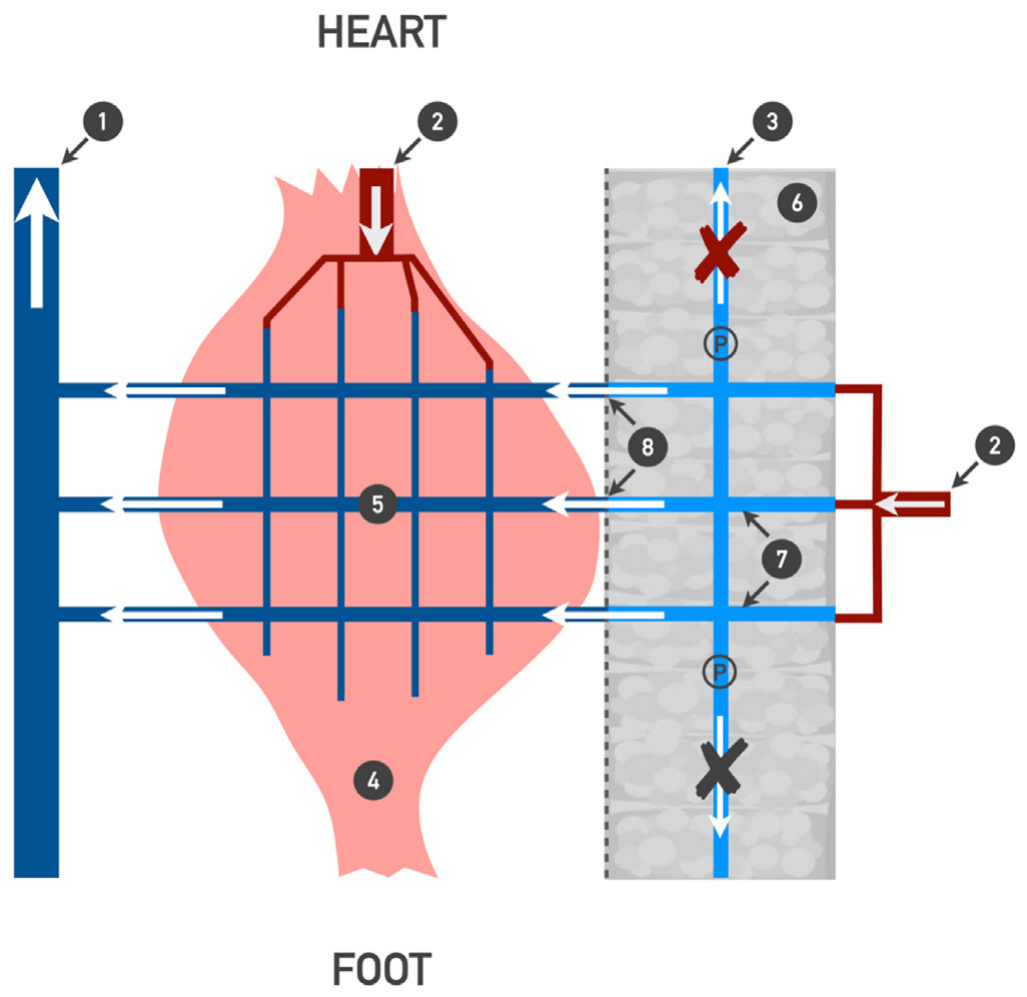
Blood outflow from the superficial venous network at the lower leg level during exercise. 1, Axis deep vein of the lower leg; 2, arterial blood flow; 3, axis superficial vein of the lower leg; 4, gastrocnemius muscle; 5, intramuscular venous network; 6, subcutaneous adipose tissue; 7, superficial venous network; 8, perforating veins. *P*, points of pressure measurement in superficial veins (ambulatory venous pressure). *Arrows*, direction of blood flow; *arrows with red cross*, observed pressure gradient prevents blood flow directed towards the heart in the superficial veins during exercise; *arrows with black cross*, competent venous valves prevent retrograde blood flow in the superficial veins.

There is general agreement that blood flow in the superficial veins is directed cranially during the muscle contraction phase of leg movements.^30^ However, it was not confirmed for natural human locomotion (walking or running). Bjordal^31^ has demonstrated that in CVD patients, the volume of blood flow in cranial direction is insignificant, and the resulting flow was directed retrogradely. The present study, conducted on normal subjects, revealed that in the GSV, the pressure gradient is directed from the thigh to the mid-calf (retrogradely) and from the ankle to the mid-calf (anterogradely) throughout the entire stride cycle at 30 stride cycles/min. As the stride cycle frequency increased, the gradient decreased. Consequently, the probability of cranial flow occurring in the GSV is negligible from the calf to thigh level, while it is presumed to occur from the ankle to mid-calf level. Therefore, it can be postulated that blood primarily flows out of the superficial venous network through the perforating veins towards the IVs during locomotion (horizontal route) (Fig 6). This horizontal route represents the major pathway for blood outflow. It can be speculated that hyperemic cranial outflow from the superficial venous network through two main axial trunks (GSV and small saphenous vein) requires excessive energy expenses due to high hydraulic resistance. The hydraulic resistance of a vessel is inversely proportional to its diameter to the fourth power and directly proportional to its length. Therefore, hydraulic resistance of the cumulative horizontal route should be considerably less owing to the total diameter of all perforating veins being significantly higher, whereas their length is much smaller than the length of the axial saphenous veins.

The absolute time of the ΔP-negative phase (when pressure in the IV is lower than pressure in the GSV) progressively decreases with the increase in stride frequency, whereas arterial blood flow increases exponentially (Fig 2, Fig 3, Fig 4 and 5, *C*). The majority of the blood in the CFA is directed towards the working muscles,^32^ and the skin and subcutaneous blood flow of the exercising leg also increase progressively.^33^ Considering that observed pressure gradient prohibits cranial blood flow in the GSV, the pressure in the superficial veins should have risen along with increasing exercise intensity. Contrary, as exercise intensity increased, the data showed a consistent level of cycle time-averaged pressure across all levels of the GSV (Fig 2). A similar pattern of the time-averaged pressure changes in the GSV at the ankle level has been previously demonstrated by Stick et al^34^ and then Eifell et al^18^ during treadmill walking and running tests. This phenomenon is likely due to the increase in CMP productivity, which compensates for the reduced blood flow time associated with the decreased ΔP-negative time and the increased arterial inflow (Fig 5). That can be supported by the observed strong relationships between *N*_*E*_, *N*_*S*_, and LBF (*N*_*S*_ vs LBF r = 0.91, *N*_*E*_ vs LBF r = 0.9; *P* < .0001; $LBF\left(f\right)=0.3e^{0.0328f}$, $\dot{NS}\left(f\right)=0.6e^{0.0325f}$, $\dot{NE}\left(f\right)=0.4e^{0.0331f}$). Therefore, during ambulation, the pressure in the superficial venous network depends upon the capacity of the muscle pump to provide output, which is proportional to the changes in arterial blood flow (Fig 6).

It follows that increased AVP (>30 mmHg) in the subjects without venous flow abnormalities should be observed when increased leg arterial blood supply is not met by the muscle pump output. The current study showed it by using another type of movement (plantar flexion) that has significantly lower minute unit power of the muscle pump (*N*_*E*_, and *N*_*S*_) uncoupled with LBF changes (Fig 5). As a result, the cycle time-averaged pressure in the GSV was 50% higher during the plantar flexion exercises than during the corresponding walking frequencies (Fig 1, *D*). Previous studies using comparable exercise tests, such as treadmill walking and plantar flexion, reported similar difference in AVP levels.^11^^,^^18^^,^^35^ Kügler et al^35^ have demonstrated that limited ankle joint mobility results in decreased calf muscle activity, ultimately leading to an increase in AVP. Impaired ankle joint mobility is strongly associated with advanced stages of chronic venous insufficiency.^19^^,^^20^^,^^36^^,^^37^ Additionally, Henry in 1948^22^ and Hickam et al in 1949^17^ have shown that in a warm environment the decline of AVP was significantly lower compared with a cold environment for standing-walking and plantar flexions exercises. They concluded it was the result of increased arterial blood supply to cutaneous and subcutaneous tissues in a hot environment.

The other muscle pumps of the lower extremity (foot and thigh) were beyond the study scope. Nevertheless, in the condition of steady-state exercise (walking, running, and plantar flexions), there was no observed significant peak of pressure in any GSV points when the foot was landing. So, it is unlikely that a significant portion of blood was ejected from the plantar venous plexus to GSV during the stance phase of the stride cycle.

### Study limitations

The main limitation of the study is the relatively small sample size. The invasive nature of the study and unknown incidence of potential complications in this specific study protocol limit the number of participants that can be possibly enrolled. However, the pressure changes showed a very consistent pattern regardless of studied subject and exercise frequency that reduces type II error probability.

The changes in pressure gradient cannot be interpreted as immediate changes in the direction of blood flow. The actual direction of the flow depends on the magnitude of the pressure gradient and the kinetic energy of the blood. The changes in pressure gradient between the IVs and superficial veins observed in this study were large in the magnitude and lasted for extended amount of time. The pressure gradient between the thigh and calf segments of the GSV, although relatively small, continued for the entire stride cycle. Therefore, both observations can be interpreted as changes in blood flow direction.

Potentially, anthropometric data (weight, height, body mass index, sex) could impact the venous pressure measurements during exercise. The study was not designed to clarify this issue. However, the consistency of the study results among enrolled participants having different anthropometrics and physical shape shows that this impact can be not so strong.

Measuring CFA blood flow volume during exercise by ultrasound is technically challenging. In this study, LBF during postexercise hyperemia was used instead for correlation with the average minute unit power of the muscle pump. It has been previously shown that there is no difference between the LBF during exercise vs an immediate postexercise LBF.^29^

It is possible that multiple catheterizations of the main saphenous tract increased hydraulic resistance for blood flow. The GSVs in study volunteers exceeded 4 mm in diameter, whereas the catheters used were 1.3 mm (18 gauge), creating less than 10% in cross-sectional area reduction, much smaller than the 50% usually required to increase flow resistance in veins. In addition, an increase in resistance should cause a progressive rise of the GSV pressure with an increase of exercise intensity, which was not observed in this study.

It is conceivable that the catheterization of the PV may have rendered it incompetent, thereby impacting AVP. The catheter had an outer diameter of 1.7 mm, which was, on average, approximately 85% of the average inner diameter of the PV (2 mm). This resulted in a high hydrodynamic resistance and a minimal impact on pressure in superficial veins.

The action of CMP during ambulation most likely results in an increase in deep venous flow. However, the present study did not include a measurement of deep venous flow, and thus the effect in question cannot be confirmed.

## Conclusion

During natural locomotion, the muscle pump acts as a flow diverter pump, redirecting the flow of blood from the superficial veins to the IVs via the perforating veins. The muscle pump is a two-component mechanism that involves the coordinated activity of the anterior and posterior calf muscle groups. The low-pressure profile observed in the superficial system (GSV) favors a suction pressure during relaxation to fill the CMP. During ambulation, the pressure in the superficial venous network depends upon the capacity of the muscle pump to provide output that is proportional to the changes in arterial blood flow. Optimal CMP function ensures that the real-time output of the muscle pump is proportional to the increased arterial blood flow during exercise, thereby preventing an increase in venous pressure in response to increased arterial inflow.

## Author Contributions

Conception and design: RT

Analysis and interpretation: RT, FL, SS

Data collection: RT, RA, PK, MB, TG, DB

Writing the article: RT, FL, SS, TG

Critical revision of the article: RT, FL, SS, RA, PK, MB, TG, DB

Final approval of the article: RT, FL, SS, RA, PK, MB, TG, DB

Statistical analysis: RT, FL, SS

Obtained funding: Not applicable

Overall responsibility: RT

## Funding

This research was funded by Dr Vladimir Denisov and at the personal expense of researchers.

## Disclosures

None.
