# Supplementary material for: The human lower leg muscle pump functions as a flow diverter pump, maintaining low ambulatory venous pressures during locomotion
Source: J Vasc Surg Venous Lymphat Disord. 2024 Oct 22;13(1):101996. doi: 10.1016/j.jvsv.2024.101996 (PMC11764322; doi:10.1016/j.jvsv.2024.101996)
Supplement: Supplementary Appendix (online only) [file mmc1.docx]

**SUPPLEMENTAL APPENDIX**

We analyze the rate of work per unit volume of blood which is associated with the change in kinetic energy of the blood flow and potential energy of the elastic stretching of veins per unit volume of blood and unit time. According to the basic principles of fluid dynamics,^1^ this value equals the pressure derivative by time

$$N=\frac{dP}{dt}=\frac{1}{V}\frac{dW}{dt},$$

Where *N* is the unit power of the muscle pump, $P$ is pressure, $V$ is volume, and $W$ is the work of the muscle pump, which is performed by the muscle pump to move a portion of the blood of volume V. For exercises with different frequencies, we compare the unit power of the muscle pump over the same time period $T$

$$T=m_{1}\tau_{1}=m_{2}\tau_{2}=\ldots$$

Where $m$ is the number of strides or plantar flexion cycles and $\tau$ is the duration of a stride cycle in an exercise.

Thus, we compare the values $N_{1}=m_{1}\frac{{dP}_{1}}{dt}$, $N_{2}=m_{2}\frac{{dP}_{2}}{dt}$, etc. The value of $N_{j}$ equals the power of the muscle pump over period $T$ in exercise *j*.

We compute the pressure derivate $\frac{dP}{dt}$ based on the measured pressure values $P$ at time moments $t_{1},t_{2},\ldots,t_{N}$ ($N$ is the total number of time moments) by the following finite difference methods:

$$\frac{dP}{dt}\left( t_{n} \right)\approx\frac{P\left( t_{n+1} \right)-P\left( t_{n-1} \right)}{t_{n+1}-t_{n-1}},n=2,\ldots,N-1$$

$$\frac{dP}{dt}\left( t_{1} \right)\approx\frac{-3P\left( t_{1} \right)+4P\left( t_{2} \right)-P\left( t_{3} \right)}{t_{3}-t_{1}}$$

$$\frac{dP}{dt}\left( t_{N} \right)\approx\frac{3P\left( t_{N} \right)-4P\left( t_{N-1} \right)+P\left( t_{N-2} \right)}{t_{N}-t_{N-2}}$$

According to the theory of numerical methods, these formulas have the second order of accuracy.^2^

The calf muscle pump during locomotion is a cyclic process including two consecutive phases, which are ejection and suction with blood owing to the work of antagonist calf muscles (gastrocnemius and anterior tibial muscle).^3^ The force of gastrocnemius concentric contraction (an increase in tension with muscle shortening) generates blood ejection. The force of anterior tibial muscle concentric contraction with parallel gastrocnemius relaxation generates very low, even negative, pressure in the intramuscular venous network of the gastrocnemius (**Supplemental Figure 1a**). The force of those muscles is reflected in the typical pressure curve of IV as segments AB and BC (**Supplemental Figure 1b**), where “A” is the point at which the gastrocnemius begins concentric contraction (heel raise moment) and “B” is the point of the highest pressure corresponding to the end of gastrocnemius contraction and the following foot take-off when anterior tibial muscle starts the concentric contraction for dorsiflexion. “C” is the point of the lowest pressure approximately corresponding to the end of anterior tibial muscle concentric contraction (foot dorsiflexion). Points A and C correspond to the local minimum of $P\left( t \right)$, while point B corresponds to the local maximum of $P\left( t \right)$. In all measurements, we observe AB is a monotonically increasing part of $P\left( t \right)$, while BC is monotonically decreasing part of $P\left( t \right)$. According to the properties of smooth functions, AB corresponds to the positive values of $\frac{dP}{dt}$, while BC corresponds to the negative values of $\frac{dP}{dt}$. According to a necessary condition for an extremum of a smooth function, points A, B, and C correspond to zero value of $\frac{dP}{dt}$. Thus, we identify $t_{A}$, $t_{B}$, $t_{C}$ by setting the first derivative of $P\left( t \right)$ to zero and finding corresponding values $t_{A}$, $t_{B}$, $t_{C}$ with the finite difference methods shown above (see the lower row of graphs in **Supplemental Figure 1b**). Thus, the work of gastrocnemius and anterior tibial muscle per cycle can be evaluated as time-averaged unit power for the periods AB (power of ejection, *N̅_E_*) and BC (power of suction, *N̅_S_*), respectively. Finally, the average unit power per minute was calculated as *Ṅ_E_ = m* N̅_E_* and *Ṅ_S_ = m* N̅_S_* (Mpa/min) for each type of exercise, where *N̅_E_* and *N̅_S_* are cycle-averaged unit power of ejection and suction, respectively, and *m* is the number of either stride or flexion cycles per minute.

1. Landau LD, Lifshitz EM. Fluid mechanics: Landau and Lifshitz: course of theoretical physics [Internet]. Vol. 6, Image Rochester NY. 1987. 539 p. Available from: <http://www>.springerlink.com/index/10.1007/b138775

2. R.J. LeVeque. Finite difference methods for ordinary and partial differential equations. 2nd ed. Philadelphia: Society for Industrial and Applied Mathematics (SIAM); 2007.

3. Tauraginskii RA, Lurie F, Simakov S, Agalarov R, Borsuk D, Khramtsov P. Calf muscle pump pressure-flow cycle during ambulation. J Vasc Surg Venous Lymphat Disord [Internet]. 2023 Jul 1;11(4):783-792.e7. Available from: <https://doi>.org/10.1016/j.jvsv.2023.04.002

**Supplemental Figure 1**

**
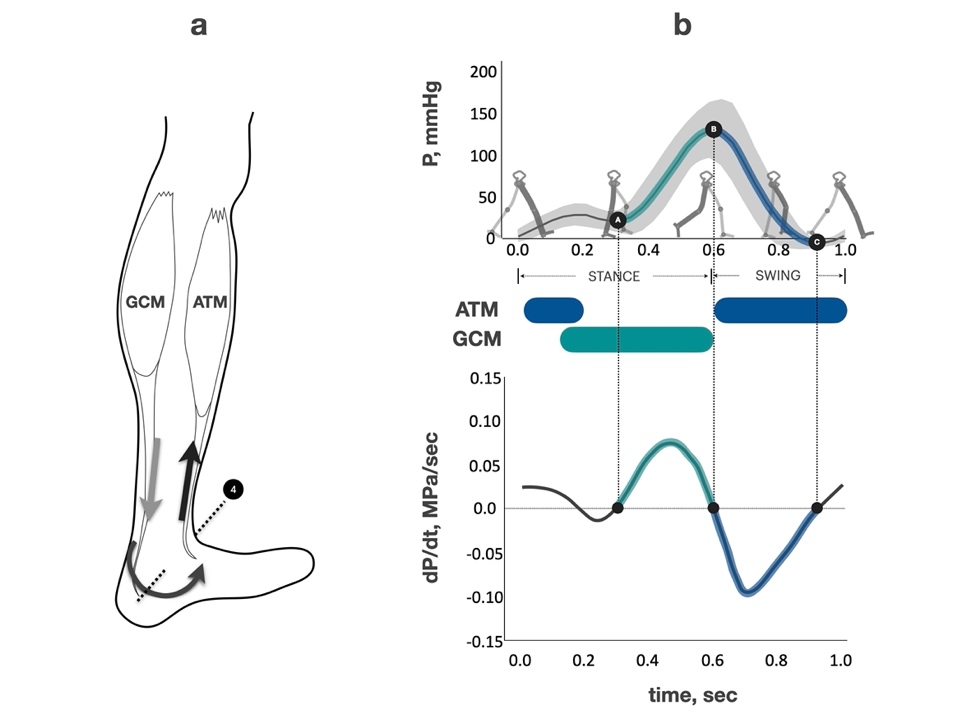
**

Legend: **Study method**

**a: *Biomechanics of dorsiflexion***

1. axis of ankle joint rotation

***GCM*** – gastrocnemius muscle

***ATM*** – anterior tibial muscle

***Arrows*** – direction of movement

**b: *Method of calf muscle pump unit power calculation***

***The top graph is the pressure changes in the intramuscular veins of the gastrocnemius across the stride cycle.***

***The bottom graph is the pressure derivative (unit power of muscle pump,*** $\frac{\boldsymbol{dP}}{\boldsymbol{dt}}$***)*** ***changes across the stride cycle.***

***A*** is the point when gastrocnemius begins concentric contraction (heel rise moment).

***B*** is the point of the highest pressure corresponding to the end of gastrocnemius contraction, and the following foot take-off when anterior tibial muscle starts its concentric contraction for dorsiflexion.

***C*** is the point of the lowest pressure approximately corresponding to the end of anterior tibial muscle concentric contraction (foot dorsiflexion).

***AB*** corresponds to the positive values of $\frac{dP}{dt}$

***BC*** corresponds to the negative values of $\frac{dP}{dt}$

***Dotted projection lines*** show that points **A**, **B**, and **C** correspond to zero value of the $\frac{dP}{dt}$

***Blue boxes*** – timeline of anterior tibial muscle activation

***Green box*** – timeline of gastrocnemius muscle activation
